# Influence of UV Radiation on Mechanical Properties of Polymer Optical Fibers

**DOI:** 10.3390/polym14214496

**Published:** 2022-10-24

**Authors:** Arnaldo Leal-Junior, Robertson Pires-Junior, Anselmo Frizera, Carlos A. F. Marques

**Affiliations:** 1 Graduation Program in Electrical Engineering, Federal University of Espirito Santo, Vitória 29075-910, Brazil; 2 I3N & Physics Department, University of Aveiro, 3810-193 Aveiro, Portugal

**Keywords:** polymer optical fibers, UV radiation, dynamic mechanical analysis, mechanical properties

## Abstract

This paper presents an analysis of the mechanical properties of different polymer optical fibers (POFs) at ultraviolet (UV) radiation conditions. Cyclic transparent optical polymer (CYTOP) and polymethyl methacrylate (PMMA) optical fibers are used in these analyses. In this case, the fiber samples are irradiated at the same wavelength, pulse time and energy conditions for different times, namely, 10 s, 1 min, 2 min and 3 min. The samples are tested in tensile tests and dynamic mechanical thermal analysis (DMTA) to infer the variation in the static and dynamic properties of such fibers as a function of the UV radiation condition. Furthermore, reference samples of each fiber (without UV radiation) are tested for comparison purposes. The results show a lower UV resistance of PMMA fibers, i.e., higher variation in the material features in static conditions (Young’s modulus variation of 0.65 GPa). In addition, CYTOP fiber (material known for its high UV resistance related to its optical properties) also presented Young’s modulus variation of around 0.38 GPa. The reason for this reduction in the moduli is related to possible localized annealing due to thermal effects when the fibers are subjected to UV radiation. The dynamic results also indicated a higher variation in the PMMA fibers storage modulus, which is around 30% higher than the variations in the CYTOP fibers when different radiation conditions are analyzed. However, CYTOP fibers show a smaller operational temperature range and higher variation in the storage modulus as a function of the temperature when compared with PMMA fibers. In contrast, PMMA fibers show higher variations in their material properties when subjected to oscillatory loads at different frequency conditions. Thus, the results obtained in this work can be used as guidelines for the influence of UV radiation in POFs not only for the material choice, but also on the limitations of UV radiation in the fabrication of the grating as well as in sensor applications at UV radiation conditions.

## 1. Introduction

The use of optical fibers in sensor applications is motivated by their compactness, lightweight, multiplexing capabilities, electrical insulation and electromagnetic field immunity [1,2]. Regarding the material properties, the optical fibers can be classified as silica and polymer optical fibers (POFs). POFs present advantages related to their material properties when compared with silica fibers, which include higher fracture toughness, flexibility in bending, lower Young Modulus, and higher failure strain and biocompatibility with the drawbacks related to higher transmission losses when compared with silica optical fibers [3]. To address this issue, reports on the fabrication of graded-index POFs [4] and their doping with different materials [5] have been made resulting in significant decreases in optical losses.

Although polymethyl methacrylate (PMMA) is the most employed material for POF manufacturing [6], there are many reports of POF fabrication with different materials, such as Zeonex [7], TOPAS [8] and polycarbonate (PC) [9]. It is also worth mentioning that cyclic transparent optical polymer (CYTOP) fibers are employed at the 1550 nm wavelength region due to their lower transmission losses at such wavelength regions when compared with other POFs [4].

The development of polymer optical fiber Bragg gratings (POFBGs) in multimode POFs has already been reported in the literature [10,11,12]. However, POFBGs are usually inscribed in single-mode POFs, such as microstructured POFs (mPOFs) that present a pattern of holes through the fiber separated by a certain pitch [13]. The limitations imposed by the time needed to write a single POFBG with the 325 nm UV laser are related to the lower photosensitivity of the POF at this wavelength when compared with lower UV wavelengths. The use of a 266 nm UV laser on the POF radiation improves the inscription time of POFBG. However, it also leads to the necessity of evaluation of the optical properties and UV radiation influence on the polymer’s mechanical properties. The careful control of the laser parameters (repetition rate and energy) indicates that a POF can be irradiated under an incubation phenomenon, for which there are no signs of polymer ablation [14].

In general, the polymers used in optical fiber development are intrinsically photosensitive [15], but undoped POFs generally need longer inscription times. Such long inscription times lead to some challenges related to the necessity of higher stability of the setup during inscription [16]. Nevertheless, the time taken for a POFBG inscription can be reduced by several orders of magnitude with the application of a 248 nm laser with low fluence and repetition rate through the phase mask technique [17]. However, the polymer is a viscoelastic material that does not present a constant response to stress or strain [18] and a creep or relaxation may be observed both in stress–strain cycles [19] and long-term tests with strain cycles applied [20]. In this case, the UV radiation in the fiber can lead to variations in the material properties that may affect its performance in sensor applications. It is worth noting that longer times for FBG inscriptions lead to longer radiation exposure, which results in variations in the mechanical properties of the fiber. In addition, for field applications of optical fiber sensors, the UV radiation from the environment can also lead to differences in the sensor responses and material properties. Furthermore, materials with higher UV resistance (such as CYTOP) need longer inscription times if the FBG inscription is performed using UV lasers.

In addition, Young’s modulus variation in the PMMA mPOF was characterized by a frequency range of 1 to 2 kHz in [21]. However, the effect of temperature and humidity on Young’s modulus variation in a PMMA mPOF also needs to be characterized, since PMMA POFs present sensitivity to such parameters [22]. Furthermore, Young’s modulus variation with the temperature for bulk PMMA POFs and their relation with frequency variations as well as the humidity is presented in [23,24]. It is also worth noting that CYTOP fiber material properties have been also analyzed in different sensor applications [25,26].

Considering this background, it is possible that the FBG inscription leads to variations in the polymer material properties due to the UV radiation in the optical fiber. As the FBG sensors are directly related to the material properties, especially the mechanical properties in physical sensors, the influence of UV radiation on the material features can lead to differences in the sensor’s responses in both static and dynamic conditions. In order to evaluate this influence, this paper presents the characterization and analysis of POFs at different UV radiation conditions. The static and dynamic mechanical properties of PMMA and CYTOP optical fibers are evaluated at different UV radiation conditions to evaluate such properties of each material, where the CYTOP has a well-known UV resistance [25] that can be used for the material’s comparison with the widely used PMMA material in POFs.

## 2. Materials and Methods

**Figure 1:**
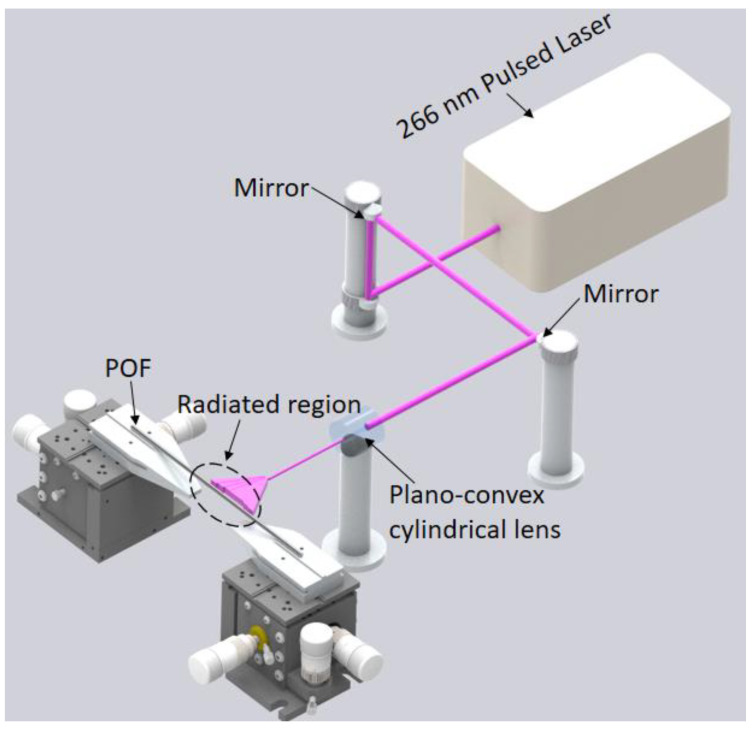
Experimental setup for UV radiation in the POF samples.

For the UV radiation on the POF samples, a pulsed Q-switched Nd:YAG laser system (LOTIS TII LS-2137U) emitting the fourth harmonic (266 nm) was employed [27]. In this case, the pulse energy is 120 µJ with repetition rate of 1 Hz, which presents a circular beam profile with diameter around 8 mm and divergence lower than 1.0 mrad. The experimental setup shown in Figure 1 also presents plano-convex cylindrical lens with effective focal length of 320 mm to focus the laser beam onto the optical fiber, where the effective spot size of the beam on the fiber surface was 8 mm wide and 30 µm high.

The POFs are positioned in the setup as shown in Figure 1 and the samples are irradiated with the 266 nm laser. Both fiber samples are subjected to abrasive removal of material to expose their cores, where it is also worth mentioning that similar procedure is performed in the samples without UV radiation for comparison purposes. In this case, there is the removal of the optical fiber cladding and overcladding using a rotary tool with a polishing sandpaper for the reduction in the surface roughness. The PMMA fiber has a core of around 980 µm with a cladding of the fluorinated PMMA material and an overcladding of polyethylene, whereas the CYTOP fiber has a 120 µm core with a cladding thickness of 20 µm and a polycarbonate overcladding. The PMMA and CYTOP samples are irradiated with constant frequency and energy, but with different radiation times. Table 1 summarizes the fabricated samples with the POF material and radiation time. UV radiation can influence the PMMA mechanical properties, which can be proportional to the radiation time. In contrast, as CYTOP is a UV-resistant material, the influence of the UV radiation in this material can be limited to the mechanical properties from thermal effects when the fiber is under UV radiation [28]. Thus, the comparison between PMMA and CYTOP fibers indicates the influence of UV radiation on each material and the UV resistance of the CYTOP.

The mechanical characterization of the samples presented in Table 1 is performed in static and dynamic conditions. The different sets of samples are analyzed through tensile tests using a universal testing machine (Biopdi, São Carlos, Brazil), where the samples are about 100 mm in length. The tests are performed with constant strain rate of 10 mm/min and the analyzed mechanical properties in the tensile tests are Young’s modulus, obtained from the slope in the stress–strain curve following the ISO 527-1:2019 Standard for tensile properties in plastics.

The POFs samples (with and without UV radiation) are positioned on the dynamic mechanical analyzer DMA 8000 (Perkin Helmer, Waltham, MA, USA). The length of the fiber samples is about 10 mm, whereas each clamp has 3 mm length. Therefore, only 4 mm of the fiber will be under test. Thus, the longitudinal uniformity of the fiber will present lower influence on the test results, since a such small portion of the fiber is under stress, temperature and frequency variations.

The Dynamic Mechanical Thermal Analysis (DMTA) is performed by applying an oscillatory load with controlled frequency and amplitude sample. One end of the fiber is fixed in the oscillatory support and the other end is fixed, i.e., without movement. For the force (stress) assessment in the sample, a load cell is positioned within the fixed support, whereas the strain is measured with a linear variable differential transformer (LVDT) sensor positioned in the oscillatory support. Furthermore, the temperature variation is performed using a heater inside the DMTA chamber and the temperature control is achieved with a temperature sensor positioned close to the sample.

The analyzed parameters in DMTA tests include the storage modulus (*E′*), loss modulus (*E″*) and relaxation time (*τ*). Using these three parameters, it is possible to determine the viscoelastic behavior of the material [29] through a combination of the storage and loss modulus is the dynamic Young’s modulus (E*) of the polymer, see Equation (1).

(1) E*=E′+iE″

It is also important to mention that the ratio between the storage and loss modulus is the loss factor tan *δ* defined in Equation (2). This parameter is related to the ratio between the dissipated energy and the storage energy per cycle of applied load.

(2) $$\tan\delta =\frac{E\prime \prime }{E\prime }$$

## 3. Results

**Figure 2:**
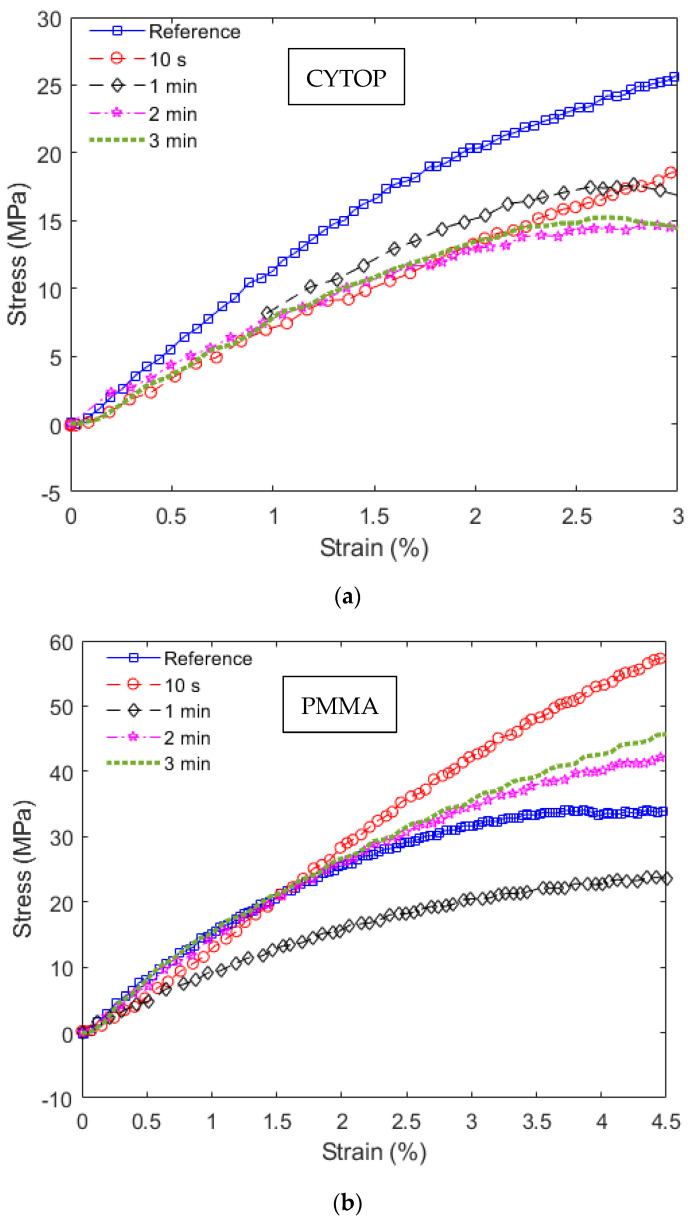
Stress–strain curves in static tests at different UV radiation conditions for (**a**) CYTOP fibers and (**b**) PMMA fibers.

The stress–strain curves obtained in the static tests are shown in Figure 2, where Figure 2a shows the results of the CYTOP fiber samples and Figure 2b presents the stress–strain curves of the PMMA fibers. In these cases, Young’s moduli of the fibers can be estimated from the linear region of the stress–strain curves. From the results in Figure 2, it is possible to observe a higher variance of Young’s moduli in CYTOP samples when compared with the ones of PMMA POFs. However, there is a reduction in Young’s modulus of each UV-radiated sample when compared to the reference one, including the CYTOP fiber. Thus, the mechanical properties of the CYTOP material are sensitive to UV radiation.

**Figure 3:**
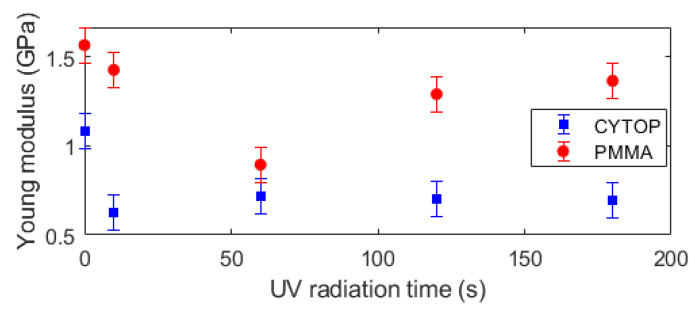
Young’s modulus of PMMA and CYTOP samples at different UV radiation times.

In general, the CYTOP fibers have lower Young’s modulus than the PMMA POF ones. However, the modulus reduction as a function of the UV radiation time is lower on CYTOP fibers when compared with PMMA samples with oscillations when the radiation time is higher than 60 s in both fiber materials, especially on PMMA fiber. In addition, there are only small variations in Young’s modulus of the CYTOP fibers when the radiation is longer than 10 s, which can indicate possible saturation of the mechanical properties variation with the UV radiation time. In order to obtain a better visualization of the mechanical property at each condition, Young’s modulus of each sample is presented in Figure 3 as a function of the UV radiation time. The results presented in Figure 3 indicate a higher variation in the PMMA’s Young’s modulus as a function of the radiation time, where there is a decrease in Young’s modulus until 60 s of UV radiation followed by a minor increase in longer radiation times. The Young’s modulus reduction obtained for all materials can be related to thermal effects in the polymer when there is UV radiation. Although all tests were performed at room temperature, the UV radiation can lead to a sharp increase in the temperature of the radiated region, which can be higher than 10 °C (depending on the radiation time and energy), which can be sufficient to minor annealing effects in the optical fiber [30]. This radiation condition can lead to a localized temperature increase in the polymer core, which can result in a local annealing effect that causes this reduction in Young’s modulus [31].

**Figure 4:**
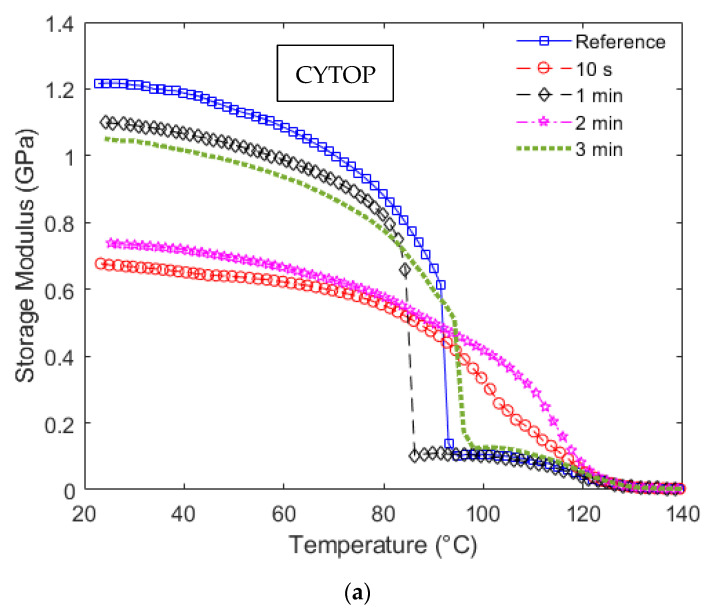

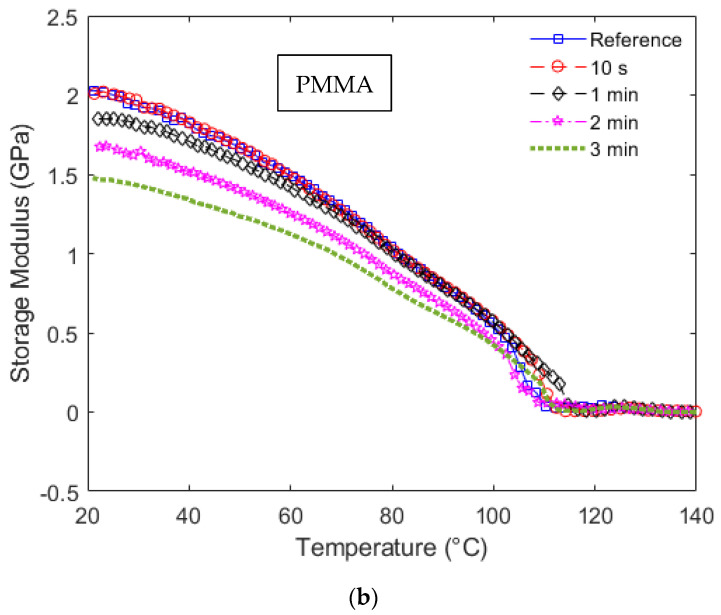
Storage modulus as a function of the temperature at each UV radiation condition for (**a**) CYTOP and (**b**) PMMA fiber.

Following the mechanical analysis of the UV irradiated POFs, the dynamic analysis performed in DMTA results in the storage modulus variation as a function of temperature and frequency. Considering the storage modulus variation as a function of the temperature, Figure 4a shows these results for CYTOP fibers, whereas Figure 4b presents the storage modulus curves for the PMMA samples.

The results of Figure 4a show a sharp decrease in the CYTOP fiber in almost all radiation conditions (except 10 s and 2 min). In general, the temperature increase leads to molecular alignment relaxation, resulting in a reduction in the material modulus [31,32]. The results show a higher variation in the CYTOP with 1 min radiation with the temperature. Considering sensing applications, this variation indicates a higher temperature cross-sensitivity that may be a disadvantage in applications with variation in both strain and temperature and compensation techniques for such effects are needed [33]. The reason for temperature cross-sensitivity increase due to storage modulus variation is related to the dependency of mechanical sensor sensitivities to the material properties, e.g., if the sensor response is proportional to the material’s Young’s modulus, variations in this parameter will lead to variations in the sensor responses. Therefore, the temperature influence can lead to cross-sensitivities and the necessity of compensating this parameter. Moreover, all CYTOP samples presented variations in the storage modulus as a function of the temperature and UV radiation time. In addition, this high variation occurs at around 80 to 100 °C for all samples in Figure 4a, which can limit the application range of such fibers with UV radiation.

The PMMA fiber results, shown in Figure 4b, indicate a linear reduction (correlation coefficient of around 0.92) in the storage moduli as a function of temperature until around 80 °C for all analyzed samples. Such linearity in the storage modulus variation is beneficial for sensor applications that involve simultaneous variation in mechanical parameters and temperature, since most of the temperature compensation techniques and methods for simultaneous measurement of temperature and strain are based on linear (or linearized) models [34]. If only the temperature range of 20 °C to around 45 °C is analyzed, the storage modulus curve of each radiation condition is parallel, which indicates that, for this specific temperature range, the dynamic mechanical properties of the PMMA POF at different UV radiation conditions can be estimated from the reference sample with only the offset of curves.

**Figure 5:**
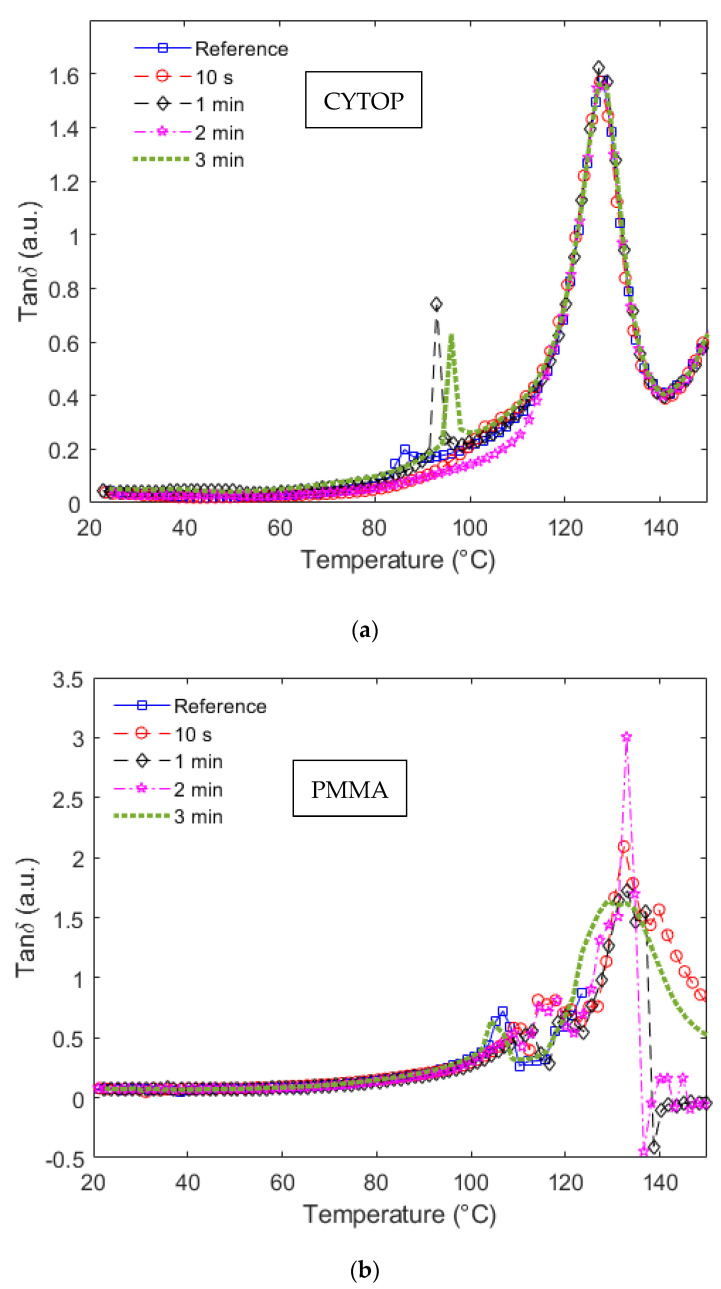
*tanδ* as a function of the temperature at each UV radiation condition for (**a**) CYTOP and (**b**) PMMA fiber.

It is also worth mentioning that the temperature at which such a sharp reduction in the storage modulus occurs indicates a glass transition temperature (*Tg*), i.e., the temperature at which the material commences rubbery behavior. In a DMTA approach, the *Tg* can be estimated from the *tanδ*, defined as the ratio between storage and loss moduli, as shown in Equation (2). In this case, the transition occurs when the loss modulus is higher than the storage modulus of the polymer, i.e., when the *tanδ* is higher than one or, more specifically, in the global peak of the *tanδ* curve as a function of the temperature. In order to verify this, Figure 5a,b show the *tanδ* as a function of the temperature for CYTOP and PMMA samples, respectively.

In Figure 5a, the peak in the curves of all samples occurs at 127.0 ± 0.5 °C, which indicates that there are no significant variations in the *Tg* of CYTOP fibers when submitted to UV radiations. Furthermore, when Figure 4a and Figure 5a are compared, there is a sharp reduction in the storage modulus of the CYTOP fibers subjected to UV radiation times of 0 min (reference sample), 1 min and 3 min (Samples 1, 3 and 5), where such behavior is also represented in the *tanδ* curves. In these cases, the *tanδ* curves show a local peak at the temperatures at which there is a sharp decrease in the storage modulus curves, see Figure 4a. Considering Samples 1, 3 and 5, it is possible to observe the sharp increase in the *tanδ* at around 86 °C, 93 °C and 96 °C for Samples 1, 3 and 5, respectively. This local peak can indicate a secondary transition temperature in the samples, which can harm their applications at such temperatures.

Similarly, some of the PMMA samples also presented this secondary transition (Samples 6, 9 and 10 related to the PMMA reference, 2 min and 3 min samples). However, this local peak is at temperatures higher than 100 °C in all analyzed cases. Moreover, the *tanδ* curves in Figure 5b presented global peaks at around 130 °C, which is higher than the ones obtained in the CYTOP fibers. This result indicates that the PMMA fibers can operate in a higher temperature range when compared with the CYTOP ones. Nevertheless, there is also a higher variation in the *Tg* (global peak of the *tanδ* curves) if the different PMMA samples are considered. A minor reduction in the *Tg* can be observed when there is UV radiation in the fiber. The maximum *Tg* of all PMMA POFs samples is around 132.4 °C, whereas the minimum value, obtained in the sample irradiated for 3 min, is around 128.9 °C. Compared with the CYTOP fiber samples shown in Figure 5a, the highest variation in the *Tg* is around 1.3 °C (maximum value is 128.0 °C and minimum value is 126.7 °C).

**Figure 6:**
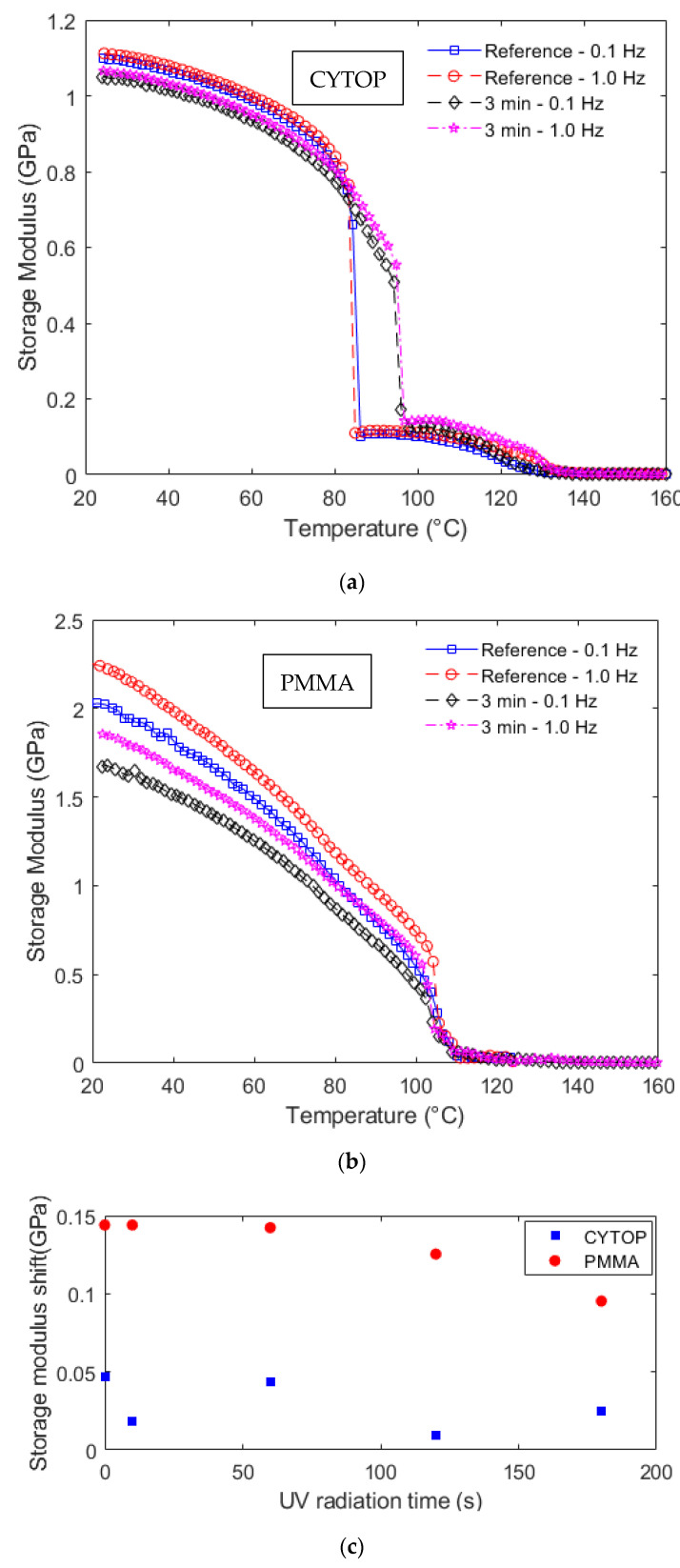
Storage modulus as a function of the temperature at different frequencies for (**a**) CYTOP and (**b**) PMMA fiber. (**c**) Storage modulus shift at 80 °C for all samples at different UV radiation times.

The frequency dependency of viscoelastic materials is anticipated from the time–temperature superposition principle. Therefore, the variations in the frequency of the oscillatory loads in the DMTA tests lead to variations in the dynamic mechanical responses of the polymers, especially the storage modulus. To evaluate this variation, Figure 6 shows the results obtained for the CYTOP and PMMA samples storage modulus as a function of temperature at different frequency conditions (0.1 Hz and 1 Hz). In this case, Figure 6a shows the storage moduli as a function of the temperature for two different frequencies considering Samples 1 and 5, where it is possible to observe a shift in the storage modulus curve with the frequency increase. Similarly, Figure 6b shows the storage moduli as a function of temperature for Samples 6 and 10 at different frequencies. In order to verify this behavior at all fiber samples, Figure 6c shows the frequency-induced shifts in all samples as a function of the UV radiation time for a temperature of 80 °C.

The comparison between Figure 6a,b indicates a larger storage modulus shift in the PMMA samples. This observation is confirmed in the results presented in Figure 6c, where there is a higher influence of the frequency on the PMMA fibers in which the storage modulus shift is around three times higher than the one obtained in the CYTOP samples. It is also worth noting that the PMMA fibers presented a downward trend on the storage modulus shift as the UV radiation time increases, whereas no obvious trend is found on the CYTOP samples, which presented a mean storage modulus shift of 0.0287 ± 0.0163 GPa. This shift was obtained when the oscillatory load frequency was increased from 0.1 to 1.0 Hz. These results indicate that the increase in the UV radiation time can reduce the frequency sensitivity of the PMMA fibers, which can be beneficial for dynamic sensor applications. In addition, the results also indicate that CYTOP fibers are preferable in dynamic applications, since the storage modulus has a smaller variation with respect to the applied frequency in the oscillatory loads.

## 4. Conclusions

This paper presented an analysis of UV radiation’s influence on POFs, namely, CYTOP and PMMA fiber mechanical properties considering both static and dynamic conditions. The analysis was performed in UV-irradiated samples with radiation times of 10 s, 1 min, 2 min and 3 min. In addition, reference samples, without any UV radiation conditions, were also tested for comparison purposes. Results show higher variation in the static and dynamic properties, namely, Young’s modulus and storage modulus, for the PMMA fibers (around 0.65 GPa variation) when compared with one of the CYTOP fibers (variation of around 0.38 GPa). Thus, it is possible to infer that the PMMA fibers have lower UV resistance when the mechanical properties are considered. However, the CYTOP fibers still presented a reduction in Young’s modulus after the UV radiation, which indicates that the UV resistance of the CYTOP fibers has a stronger relation to their optical properties than their mechanical ones. The reason for this reduction in the moduli is related to possible localized annealing due to thermal effects when the fibers are subjected to UV radiation.

The dynamic analysis also indicated the temperature and frequency responses of the materials at different UV radiation conditions. The results indicated a higher temperature dependency and lower temperature range of the CYTOP fibers at each UV radiation condition. In contrast, PMMA fibers presented higher storage modulus variation as a function of the applied frequency. Therefore, the PMMA fibers are preferable in applications with temperature variation conditions, whereas the CYTOP fibers are preferable at frequency variation conditions. Future works include sensor applications for UV detection and the use of such POFs in applications with high radiation conditions.

## Figures and Tables

**Table 1:** Samples’ material and UV radiation time.

Sample ID	Material	UV Radiation Time
CYTOP0	CYTOP	0 s
CYTOP10	CYTOP	10 s
CYTOP60	CYTOP	1 min
CYTOP120	CYTOP	2 min
CYTOP180	CYTOP	3 min
PMMA0	PMMA	0 s
PMMA10	PMMA	10 s
PMMA60	PMMA	1 min
PMMA120	PMMA	2 min
PMMA180	PMMA	3 min

## Data Availability

Not applicable.
